# Pseudotumor of Ciliary Body

**DOI:** 10.1155/2014/458683

**Published:** 2014-10-16

**Authors:** Mary Varghese, Raghavendra Ramappa, Sripathi Kamath

**Affiliations:** Department of Ophthalmology, St. John's Medical College, Bangalore 560034, India

## Abstract

Orbital pseudotumor is a benign disease involving the orbital structures. Pseudotumor of the ciliary body is rare. We present a case of a 27-year-old male who presented with gradual visual loss, pain, and redness in his left eye. On examination he was found to have a yellowish white mass at the periphery of anterior chamber in his left eye and ultrasound biomicroscopy (UBM) revealed a ciliary body mass in the same eye. He was treated with systemic steroids, which was tapered over a period of 8 weeks. His symptoms improved and the ciliary body mass disappeared with no recurrence over the next 6 months. UBM is an important diagnostic tool for diagnosing ciliary body mass. Early diagnosis and prompt treatment with systemic steroids may help resolve pseudotumor of the ciliary body.

## 1. Introduction

Orbital pseudotumor is a benign disease process affecting the orbital tissues. The precise aetiology of pseudotumor is not known but immune mediated causes, infectious causes, and other causes have been postulated. Among the structures in the orbit, involvement of the ciliary body is very rare.

## 2. Case Presentation

**Figure 1:**
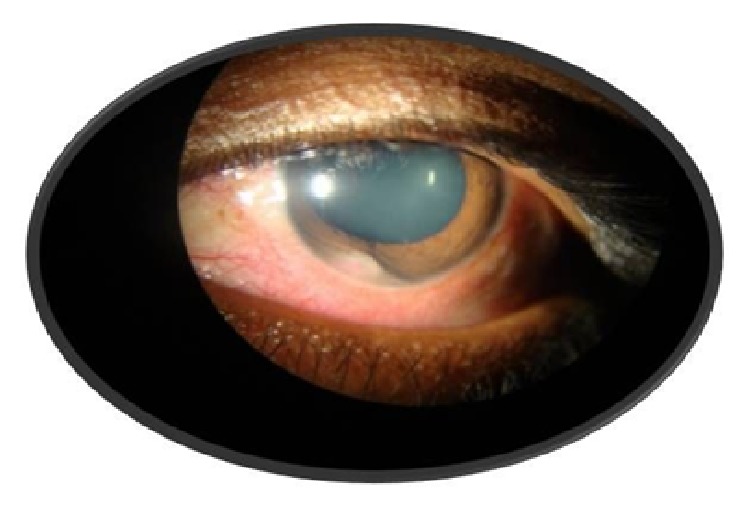
Slit lamp picture of left eye showing mass at 7 o'clock in the periphery of the anterior chamber.

**Figure 2:**
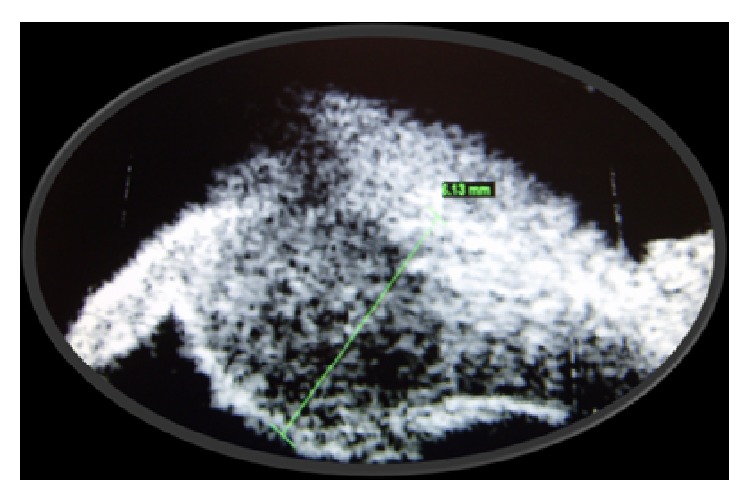
UBM picture showing the ciliary body mass.

We present a case of a 27-year-old male who presented with gradual visual loss, pain, and redness in his left eye for 2 weeks. On examination his best corrected visual acuity of the right eye was 20/20 and of the left eye was 20/50. The left eye showed circumcorneal congestion and 2+ cells. A yellowish white mass was seen from 7 to 8 o'clock position in the periphery of the anterior chamber (Figure 1) and the chamber was shallow in the same area. There was also peaking of the pupil to the same area with sluggish pupillary reaction. Anterior segment examination of the right eye was within normal limits. Fundus examination of both eyes was normal and the intraocular pressure was 15 mm Hg in both eyes. UBM of the left eye showed a ciliary body mass at 7 o'clock position measuring 6.1 mm in diameter (Figure 2).

**Figure 3:**
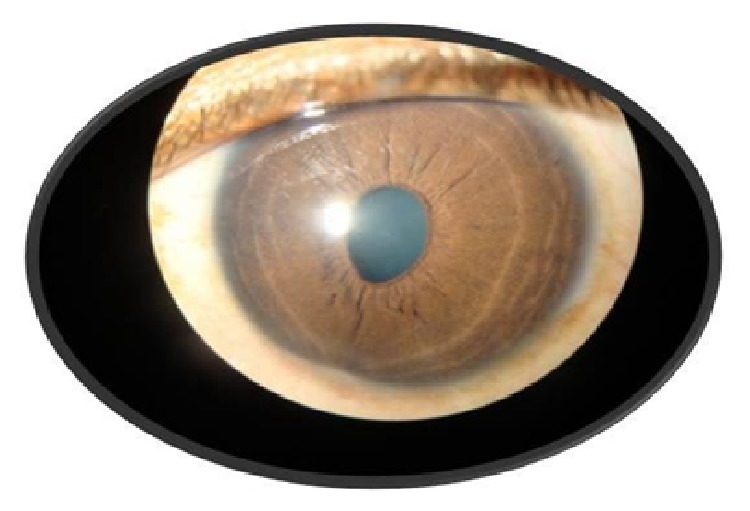
Slit lamp picture showing partial resolution of the mass at 2 weeks.

**Figure 4:**
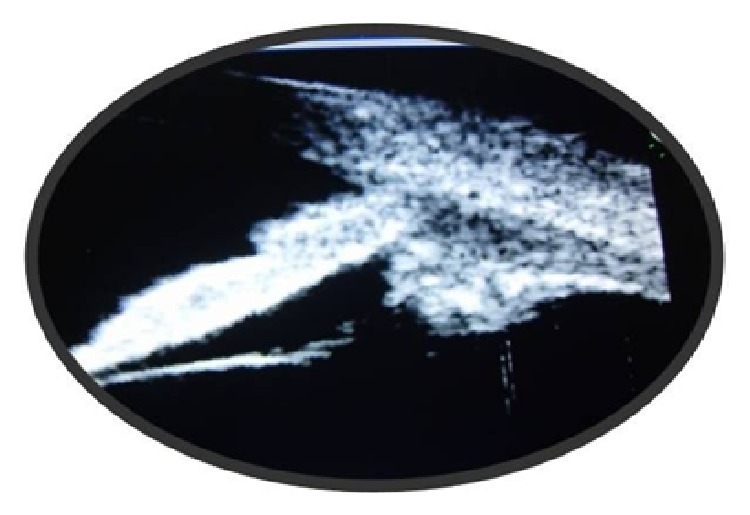
UBM picture showing partial resolution of the mass at 2 weeks.

**Figure 5:**
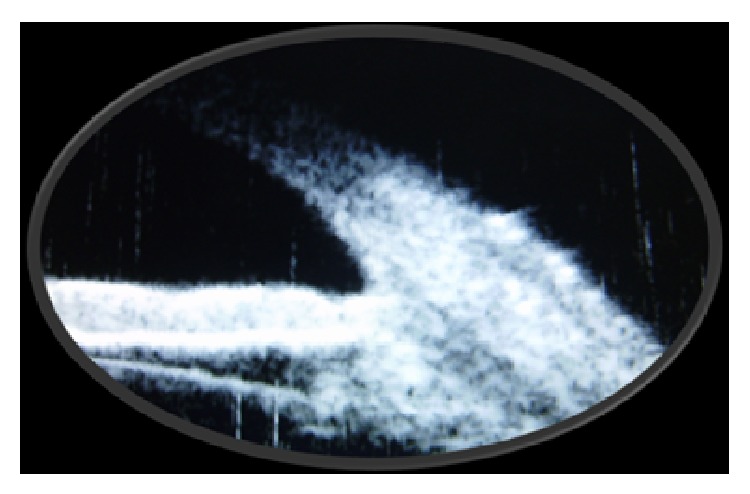
UBM picture showing near total resolution of the mass at 6 weeks.

Apart from a raised erythrocyte sedimentation rate (27 mm/hr), all the investigations including complete blood count, fasting blood sugar, antinuclear antibody, fluorescent treponemal antibody absorption, angiotensin converting enzyme levels, Mantoux test, chest X-ray, and urine microscopy were within normal limits. He was treated with oral prednisolone 1 mg/kg for 2 weeks followed by gradual tapering of the dose over the next 6 weeks. His symptoms gradually improved and the ciliary body mass also decreased in size both clinically and in the UBM images (Figures 3, 4, and 5). There was no recurrence in the next 6 months after which the patient was lost to follow-up.

## 3. Discussion

Orbital pseudotumor was described by Birch-Hirschfeld in 1930. This disease is characterized by idiopathic nonspecific inflammation of the orbit. Histopathological classification of orbital pseudotumor includes lymphoid, granulomatous, and sclerosing types. There is evidence of the first two types getting transformed to sclerosing type in the end stage of the disease [1]. A calcifying type of orbital pseudotumor, though very rare, has also been described [2].

Pseudotumor of the ciliary body is rare. It is more commonly seen as ciliary body involvement in orbital pseudotumour. Uy et al. reported a case of sclerosing inflammatory pseudotumor of the eye, which also had a ciliochoroidal mass. Choroidal biopsy in this case revealed nongranulomatous inflammation [3]. Ryan Jr. et al. reported a case of bilateral inflammatory pseudotumor of the ciliary body in 1971. To our knowledge, that is the only case reported of isolated pseudotumor of the ciliary body. A direct biopsy of the ciliary body was taken in their case which showed nonspecific inflammatory process, consisting of numerous lymphocytes, plasma cells, and histiocytes [4]. A careful fine needle aspiration biopsy may also be tried for ciliary body mass as done in a case of uveitis with iris mass, which may help in the diagnosis [5].

Ciliary body pseudotumor shows a good response to treatment with systemic steroid therapy [4]. Different types of orbital pseudotumors have shown varying treatment responses. The granulomatous type of orbital pseudotumor has a good response to systemic steroid therapy whereas the lymphoid type responds well to radiotherapy. The sclerosing type shows poor response to steroid and radiation therapy. Therefore it is important to treat orbital pseudotumor at the early stage [1, 6, 7]. In cases refractory to systemic steroid treatment, various therapeutic alternatives have been tried [2, 7].

Our patient presented with signs of ocular inflammation and a ciliary body mass. Inflammatory causes like tuberculosis or sarcoidosis were considered. Other differential diagnosis included neoplasia, lymphoma, and fungal granuloma of the ciliary body [4, 5]. Since all the tests except erythrocyte sedimentation rate were within normal limits and the patient responded well to systemic steroid therapy, we considered the diagnosis of pseudotumor of the ciliary body of the granulomatous type. Isolated cases of pseudotumor of ciliary body have rarely been reported. UBM is an important tool for diagnosing ciliary body mass and for monitoring its progression and resolution. We stress the importance of early diagnosis and prompt treatment with systemic steroids for complete resolution of the pseudotumor without further complications.
